# Potential immunological consequences of pharmacological suppression of gastric acid production in patients with multiple sclerosis

**DOI:** 10.1186/1741-7015-10-57

**Published:** 2012-06-07

**Authors:** Sangita Biswas, Stephen H Benedict, Sharon G Lynch, Steven M LeVine

**Affiliations:** 1 Department of Molecular and Integrative Physiology, University of Kansas Medical Center, Kansas City, KS, USA; 2 Institute of Pediatric Regenerative Medicine, University of California Davis, Sacramento, CA, USA; 3 Department of Molecular Biosciences, University of Kansas, Lawrence, KS, USA; 4 Department of Neurology, University of Kansas Medical Center, Kansas City, KS, USA

**Keywords:** Antacid, autoimmune, dyspepsia, experimental autoimmune encephalomyelitis, GERD, histamine receptor 2 antagonists, multiple sclerosis, proton pump inhibitor

## Abstract

Corticosteroids are standard treatment for patients with multiple sclerosis experiencing acute relapse. Because dyspeptic pain is a common side effect of this intervention, patients can be given a histamine receptor-2 antagonist, proton pump inhibitor or antacid to prevent or ameliorate this disturbance. Additionally, patients with multiple sclerosis may be taking these medications independent of corticosteroid treatment. Interventions for gastric disturbances can influence the activation state of the immune system, a principal mediator of pathology in multiple sclerosis. Although histamine release promotes inflammation, activation of the histamine receptor-2 can suppress a proinflammatory immune response, and blocking histamine receptor-2 with an antagonist could shift the balance more towards immune stimulation. Studies utilizing an animal model of multiple sclerosis indicate that histamine receptor-2 antagonists potentially augment disease activity in patients with multiple sclerosis. In contrast, proton pump inhibitors appear to favor immune suppression, but have not been studied in models of multiple sclerosis. Antacids, histamine receptor-2 antagonists and proton pump inhibitors also could alter the intestinal microflora, which may indirectly lead to immune stimulation. Additionally, elevated gastric pH can promote the vitamin B12 deficiency that patients with multiple sclerosis are at risk of developing. Here, we review possible roles of gastric acid inhibitors on immunopathogenic mechanisms associated with multiple sclerosis.

## Introduction

The use of medications to reduce acid production in the stomach has become an increasingly routine practice in patient care. Chronic pain, discomfort and swallowing problems associated with gastroesophageal reflux disease (GERD) or peptic ulcer disease are widespread in the population. Use of acid-reducing compounds has become more common and people have begun taking them routinely for heartburn or dyspepsia. Non-steroidal anti-inflammatory drugs and other medications can cause dyspepsia and peptic ulcers that can be associated with increased acid secretion [1]. Corticosteroids that are administered to patients with multiple sclerosis (MS) to promote the resolution of acute relapses [2] can also cause dyspeptic pain in the upper abdomen associated with increased gastric acid secretion [3]. Gastric disturbances are regularly managed with a histamine receptor-2 (H2R) antagonist, proton pump inhibitor (PPI) or an antacid. These agents can be administered prophylactically or in response to dyspeptic pain when the patient is receiving a course of high dose corticosteroids [2,4-6]. Patients with MS also can take these drugs as part of their daily routine due to ongoing dyspepsia, GERD or concomitant illness. Many of these agents are prescribed by the patients' primary care physician and are available as over-the-counter medications for indigestion or related conditions. These interventions are generally considered to be safe. We propose that the use of these agents could have unintended consequences on the disease process in patients with MS, and perhaps in other autoimmune conditions.

H2R, PPIs and antacids may directly or indirectly influence the inflammatory response in patients with MS. H2R antagonists (for example, cimetidine, famotidine, nizatidine and ranitidine) act in the stomach by decreasing the basal and food-stimulated parietal cell acid secretion. H2R antagonists also act on other cell types, including endothelial cells at the blood-brain barrier (BBB), mast cells and cells of the immune system (for example, T-lymphocytes, monocytes and dendritic cells (DCs)), that play central roles in orchestrating immune-mediated pathology in MS. Although histamine release can mediate acute inflammatory events, it can also influence chronic inflammatory states [7], and evidence suggests that activation of H2R suppresses the immune response. Thus, the effects of H2R antagonists could lead to enhancement of a proinflammatory state that could result in increased disease activity in patients with MS. PPIs (for example, lansoprazole, omeprazole, dexlansoprazole, esomeprazole, pantoprazole and rabeprazole) irreversibly inhibit the H^+^/K^+ ^ATPase in parietal cells, which is used to pump protons into the gastric lumen. In addition, these inhibitors can act on other cell types including cells of the immune system. Unlike H2R antagonists, PPIs might promote immune suppression. Antacids, H2R antagonists and PPIs all result in an increased gastric pH. A prolonged elevation in gastric pH can lead to increased levels of bacteria in the stomach and small intestine, which in theory could aggravate inflammation in patients with MS. In this review, we will compare the immunological effects of different modalities directed at suppressing gastric acid, and discuss the potential implications for the disease process in MS.

### Histamine receptor-2 antagonists

Histamine levels in the cerebrospinal fluid (CSF) are higher in patients with relapsing-remitting MS and progressive MS compared with control patients [8,9]. Activation of histamine receptors can stimulate both pro- and anti-inflammatory pathways, which are mediated through the differential activation of the four G-protein coupled receptors, histamine receptor-1 (H1R), H2R, H3R and H4R [7,10]. In genetically manipulated mice unable to make histamine [11], or in mice deficient in histamine-producing mast cells [12], clinical and pathological signs of experimental autoimmune encephalomyelitis (EAE), an animal model of MS, are significantly more severe than in wild-type mice with EAE. This suggests an overall effect of histamine towards limiting autoimmune brain inflammation, which is in contrast to the role of histamine in other inflammatory conditions, such as an allergic response. Because activation of H2R appears to be a key mechanism for histamine-mediated immunosuppression, it raises the question of whether H2R antagonists aggravate disease activity in patients with MS. H2R is expressed by a variety of cells, including endothelial and systemic inflammatory cells [7], and H2R is expressed in EAE by CNS inflammatory infiltrates and possibly microglia and astrocytes [13]. Because H2R antagonists have been shown to gain access to the brain [14], they can exert effects on inflammatory cells within the CNS as well as systemically.

#### Histamine receptor-2 activation promotes a T helper cell 2 response

Active disease in MS is generally thought to be associated with an overactive T helper cell (Th) 1 response and an underactive Th2 response. For example, peripheral blood mononuclear cells from patients with MS secrete increased amounts of proinflammatory cytokines associated with the Th1 response (for example, IFN-γ, IL-12 and TNF-α) and release reduced amounts of the anti-inflammatory cytokine IL-10, associated with Th2 and regulatory T cell activity [15-17]. In contrast, immune tolerance and/or disease remission is associated with an upregulation of the Th2 cytokines (for example, IL-4 and IL-10) and TGF-β in rodents with EAE [18-22]. The elevated EAE disease activity observed in mice deficient for histamine production was postulated to be owing to the absence of suppression via H2R activation, resulting in an increased Th1 response [11]. Activation of H2R by dimaprit, a selective H2R agonist, was found to reduce clinical and pathological signs of disease severity in EAE (such as ataxia and CNS macrophage accumulation) [23] and lessen encephalitogenic T cell responses [24]. Conversely, using cimetidine to block H2R during EAE induction in guinea pigs led to a greater incidence of disease when compared with the incidence in guinea pigs given saline [25]. Cimetidine also promoted a Th1-mediated delayed type hypersensitivity reaction, an inflammatory state with some similarities to EAE [25-27].

*In vitro *studies demonstrate that H2R agonists mimic the actions of histamine [28], which inhibits the secretion of proinflammatory cytokines and stimulates the production of anti-inflammatory cytokines in human peripheral blood mononuclear cells [28-31] (Table 1). Furthermore, the effects induced by histamine were primarily mediated by H2R, evidenced by the fact that these effects were blocked by cimetidine [29,30,32]. In addition, the H2R mediates suppression of TNF-α production by mast cells [33]. Thus, histamine, via stimulation of H2R, can result in a shift of Th1/Th2 balance toward Th2-dominance (Table 1). Taken together, these studies raise the question - does selective H2R antagonism negatively influence an autoimmune state by promoting Th1 responses?

**Table 1:** Examples of immune effects induced by histamine or histamine receptor-2 agonists

Agent	Organism/cell type	Response	Reference
Dimaprit	Mice	Attenuates experimental autoimmune encephalomyelitis disease activity	[23]
Dimaprit Histamine	Mouse activated T cells	Suppresses T cell proliferation, IL-6, IL-10, IL-17 and IFN-γ production	[24]
H2 agonist Histamine (reversed by H2R antagonist)	Human neutrophils	Decreases neutrophil chemotaxis response	[44]
H2 agonist Histamine (reversed by H2R antagonist)	Human T cells	Decreases T cell proliferation	[44]
H2R agonists Histamine (reversed by H2R antagonist)	Human peripheral blood mononuclear cells	Inhibits secretion of IL-1 and IL-12, and stimulates production of IL-10	[28,29,31]
Histamine (reversed by H2R antagonist)	Human peripheral blood mononuclear cells	Inhibits secretion of TNF-α	[30]
Histamine (reversed by H2R antagonist)	Human DCs	Suppresses IL-12 production following lipopolysaccharide stimulation of DCs	[73]
Histamine (reversed by H2R antagonist)	Human DCs	Promotes Th2 response, that is, upregulation of Th2 chemokine production, by immature DCs	[74]
Histamine (reversed by H2R antagonist)	Rat mast cells	Suppresses TNF-α production	[33]
Histamine (reversed by H2R antagonist)	Human umbilical vein endothelial cells	Stimulates production of IL-6	[39]

DC: dendritic cell; H2R: histamine receptor-2; IFN-γ: interferon gamma; IL: interleukin; Th: T helper cell; TNF-α: tumor necrosis factor alpha.

#### Histamine receptor-2 activation suppresses proinflammatory T cell responses

Activation and trafficking of T cells into the CNS are important steps in MS pathogenesis. In fact, drugs that target these steps (for example, copolymer 1, fingolimod and natalizumab) reduce the severity and frequency of clinical relapses in MS [34-36]. H2R-mediated actions may represent an intrinsic mechanism that self-limits T cell activation, proliferation and trafficking, particularly in the setting of autoimmunity. For example, *in vitro *administration of histamine or an H2R agonist inhibits proliferation and IFN-γ production by mouse T cells activated against an encephalitogenic peptide used for EAE induction [24].

In an intravital microscopy model mimicking the early stages of inflammation in EAE, both H1R and H2R activation reduced the ability of myelin autoreactive T cells to adhere to inflamed brain vessels *in vivo*, which is a crucial step in the development of MS [24]. In an allergic model, H2R activation led to downregulation of leukocyte infiltration into the inflamed tissue [37]. Some studies suggest that H2R may promote BBB leakage while H1R may suppress it [38], although H2R or H1R activation are associated with an increase in endothelial cell production of IL-6 [39], and IL-6 may act to promote the maintenance of the BBB [40,41].

If H2R activation leads to the suppression of the T cell responses, then selective blockage of H2R has the potential to promote the T cell immune response. Indeed, antagonism of H2R, independent of altering histamine levels, causes immune stimulation and amplification of an existing inflammatory event (Table 2). In studies on human or mouse cells, cimetidine enhances mitogen-stimulated lymphocyte activation [42,43], reduces histamine-induced suppression of T cell proliferation [44], reduces the histamine-activated suppressor T cell response in the presence or absence of mitogen [45,46], facilitates the conversion of monocytes to macrophages [47] and reverses the histamine-induced suppression of proinflammatory cytokine synthesis [29,30,32] (Tables 1 and 2). Cimetidine increased antibody-dependent cellular cytotoxicity of T cells from patients with MS against primary rat oligodendrocytes [48]. Cimetidine inhibits regulatory T cell-like activity [49] and enhances the inflammatory response, to a DNA vaccine for example, by promoting humoral and T cell-mediated responses and inducing IL-12 production while inhibiting the production of anti-inflammatory cytokines [50]. Cimetidine can also increase antibody production and proliferation of mitogen-stimulated splenocytes in response to an immunogen [51]. Ranitidine causes immune activation in patients with a head injury [52], reverses surgery-induced immunosuppression [53-55] and was reported in a case study to exacerbate lymphocytic colitis [56]. In addition, H2R are present on basophils and mast cells and function to suppress the release of histamine and proinflammatory cytokines [33,57]. Together, these data support an immunostimulatory role of H2R antagonists, which is likely due to the blockage of H2R-mediated suppression pathways (Tables 1 and 2).

**Table 2:** Examples of immune effects by histamine receptor-2 antagonists

Agent	Cell/whole animal	Response	Reference
Cimetidine	Guinea pigs	Increases activity of delayed type hypersensitivity and experimental autoimmune encephalomyelitis incidence	[25]
Cimetidine	Mouse T cells	Inhibits induction of T suppressor cells	[45]
Cimetidine	Mouse splenocytes	Increases antibody production, and proliferation of mitogen-activated splenocytes in response to tetanus toxoid	[51]
Cimetidine	Mouse T cells	Inhibits regulatory T cell-like activity	[49]
Cimetidine	Human T cells	Reduces suppressor T cell response	[46]
Cimetidine	Human lymphocytes	Increases the mitogen-activated T cell response	[42,43]
Cimetidine	Human DCs	Increases the capacity of antigen presentation by DCs from immunosuppressed cancer patients	[75]
Ranitidine	Human T cells	Increases CD4+ T cells and mitogen-stimulated IFN-γ production from patients with head injury	[52]
Ranitidine	Human monocytes, neutrophils, natural killer cells, delayed type hypersensitivity	Reverses surgery-induced immune suppression	[54,55]

DC: dendritic cell; IFN-γ: interferon gamma.

#### Histamine receptor-2 activation polarizes dendritic cells and monocyte function towards a T helper cell 2 response

DCs are professional antigen presenting cells that specialize in the uptake of antigens and their transport from peripheral tissues to the lymphoid organs. They can also migrate into the CNS and/or differentiate from microglia [58,59] and can be present in the CSF [60]. Due to their ability to stimulate naïve T cells, DCs have a central role in the initiation of a primary immune response. Emerging data indicate that DCs play an important role in the initiation of autoimmune attacks in EAE and MS. Specifically, DC-derived cytokine signals are involved in the differentiation and proliferation of autoreactive T cells.

The profile and levels of cytokines secreted by the stimulating DCs determine whether a naïve T cell will become a Th1, Th17 or Th2 cell. Th1 CD4+ T helper cells secrete proinflammatory cytokines such as IL-1, IFN-γ and TNF-β, while Th2 type CD4+ T cells secrete IL-4, IL-5, IL-13 and granulocyte colony stimulating factor. Th1 cells and Th17 cells, which secrete IL-17, promote inflammation in MS [61,62]. Experimental studies have shown that DC-derived signals are critical for recruiting and maintaining the activity of Th1 and Th17 cells [63]. During disease activity or relapse in MS, there is a higher proportion of circulating DCs that secrete IL-12 and IL-23 [64,65]. Increased secretion of IL-12 and IL-23 from DCs, in turn, coincides with significant increases in pathogenic Th1 [66] and Th17 [67,68] activity, respectively. Thus, during relapses, Th1 and Th17 cells are overactive and Th2 activity is downregulated. Conversely, during periods of disease remission, presumably a shift in DC-derived signals promotes a reduction of the Th17 cell number to low levels [69], and CD4^*+ *^cells polarize into effector IL-4 and IL-10 producing Th2 cells, resulting in an overall anti-inflammatory environment [66,70].

Histamine influences the profile of cytokine production by maturing DCs [71]. H2R seems to play a dominant role in the regulation of DC function [71] as multiple DC subsets express high levels of H2R, whereas H1R and H4R are differentially expressed [72]. Activation of H2R on DCs results in polarizing the DCs towards a Th2-promoting environment via suppression of IL-12 production [71,73] and an increase in IL-10 synthesis [71,72]. Cimetidine has been shown to block the effects of histamine in regulating IL-12 production and Th2 polarization [73,74]. A recent study also showed that H2R activation led to suppression of blood monocyte-derived CD1a^+ ^cells, a subset of DCs possessing greater inflammatory properties than the CD1a^- ^subset, and famotidine was able to block this action [72].

Direct regulation of DC function by H2R antagonists has not been shown in MS. However, cimetidine was found to increase the antigen presenting capacity and possibly IL-12 secretion of DCs isolated from immunosuppressed patients with colorectal cancer [75]. This implied unmasking of suppressed DC function by cimetidine in cells from these patients. In MS, glucocorticoids and INF-β can reduce IL-12 secretion by immature human DCs [76,77], which raises the possibility of a reversal of DC suppression by cimetidine in patients with MS similar to immunosuppressed patients with colorectal cancer. The actions of cimetidine in patients with cancer were not necessarily solely mediated by H2R, since similar effects were not seen with famotidine [75].

#### Implications of histamine receptor-2 antagonists for patients with multiple sclerosis

Although H2R antagonists have the potential to interfere with immunosuppressive pathways, it is uncertain whether they affect the disease course in patients with MS. The findings from EAE studies supporting a role for H2R antagonists in disease progression might not translate to MS, that is, the effects of H2R antagonists could have different effects between mice and humans. Furthermore, there are several competing factors that dictate whether the immune response will become activated to promote pathology in MS, and histamine is only one of many mediators influencing the immune balance and pathogenic course. Thus, it is possible that, in the overall scheme of a complex disease, blockage of immunosuppression pathways via H2R antagonists does not influence proinflammatory conditions or counteract the immunosuppressive properties of corticosteroids. Acute exacerbation of disease activity following the ingestion of H2R antagonists is unlikely; otherwise several reports would have been expected describing these events. However, the possibility that H2R blockers promote a general increase in disease activity remains, because some pathology can be clinically silent in MS [78,79] and the compounding effects of multiple lesions may take years to impact on the clinical presentation of MS [80]. Although magnetic resonance imaging (MRI) scans can often show increased activity in the face of stable clinical features, the reverse is also true; in other words, gradual clinical change is often not apparent by MRI. Thus, H2R antagonists could aggravate ongoing pathology at a subclinical level or below the detection limits of MRI. Furthermore, given the range of histamine responses in the system, and the number of common drugs that exert some influence on the histamine pathways, H2R antagonists might influence disease activity only under a specific set of conditions or only in concert with other medications, thus making the effects difficult to recognize. To complicate matters further, some outcome measures revealed a difference only with one H2R antagonist but not with a second antagonist [43,75]. With the large number of patients taking H2R antagonists, a small, but possibly significant effect could easily be missed.

A few cases of increased autoimmune responses have been reported with these agents. These have included one case of autoimmune hepatitis in a patient with MS associated with rechallenge of ranitidine [81], new skin lesions in a patient with systemic lupus erythematosus associated with cimetidine [82], lymphocytic infiltration in patients with breast cancer associated with famotidine [83], and exacerbation of psoriasis associated with H2R antagonists [84].

### Proton pump inhibitors

PPIs are routinely used to treat acid-peptic disorders. They act by blocking gastric acid secretion via inhibition of the H^+^/K^+ ^ATPase, the proton pump of the gastric parietal cells [85]. PPIs can also act on monocytes, neutrophils and endothelial cells with the result being amelioration of the immune response [86,87]. Omeprazole [87] and possibly other PPIs cross the BBB. PPIs may block the activity of reactive oxygen species [88], which are thought to promote disease activity in the CNS of patients with MS [89]. The roles of PPIs in MS or in an animal model of MS have not been adequately studied but, as discussed below, it is theoretically possible that their action favors a suppressive role on disease activity.

#### Proton pump inhibitors can cause immune suppression

Several *in vitro *and *in vivo *studies have shown that PPIs can exert anti-inflammatory effects unrelated to the inhibition of gastric acid production [90]. These anti-inflammatory effects are seen via their anti-oxidants activity, cytokine modulation and ability to alter the expression of adhesion molecules via direct action on inflammatory cells such as neutrophils, monocytes and endothelial cells [88]. These effects can persist even after short-term delivery. As mentioned in the previous sections, altered cytokine secretion and adhesion molecules expressions in inflammatory cells play important roles in MS pathogenesis. Thus, it is possible that the anti-inflammatory properties of the PPIs may contribute to the beneficial actions of other anti-inflammatory or immunomodulatory drugs when administered concurrently in MS.

#### Proton pump inhibitors suppress inflammatory responses by neutrophils and peripheral blood monocytes

Neutrophils have been suggested to promote disease activity in EAE and MS [91-96]. In EAE, neutrophils have been detected in CNS inflammatory infiltrates [93,94] and neutrophil depletion ameliorated EAE activity [94]. Neutrophils have been postulated to induce BBB leakage during the development of EAE [96] and may be involved with the onset of axonal pathology [95]. The role of neutrophils in MS is less clear. They have been suggested to be participants in early disease development in the CNS [95], but may not be present in later stages. In relapsing-remitting MS, peripheral neutrophils are in a primed state, which could lead to enhanced activation after infection. Elevated effector mechanisms by neutrophils in relapsing-remitting MS include increased degranulation, elevated oxidative burst and higher levels of neutrophil extracellular traps [97].

PPIs suppress the production of reactive oxygen species by neutrophils and monocytes in culture, lessen their expression of adhesion molecules, and reduce their interactions with endothelial cells [86,88,98-101], which is necessary for entry into the CNS. Notably, medications that interfere with cell adhesion to the endothelium are used to suppress the occurrence of MS relapses, for example, natalizumab [35]. In addition, lansoprazole reduced the *in vitro *production of the proinflammatory cytokines TNF-α and IL-1β by peripheral blood monocytes [102]. By contrast, cimetidine blocked the inhibition of neutrophil chemotaxis induced by histamine [44].

#### Proton pump inhibitors can reduce the inflammatory state of microglia

Since some PPIs like omeprazole can rapidly penetrate the BBB [87], they would have the potential to interact with microglial cells. Activation of microglial cells may play an important role in the regulation of autoimmune inflammation in EAE and MS [103,104]. Activated microglia are thought to exert toxicity towards neurons via the production of potentially neurotoxic molecules such as proinflammatory cytokines and superoxide radicals [105]. For example, lipopolysaccharide (LPS)- and IFN-γ-stimulated human microglia show significant toxicity towards neurons in culture [106]. However, when LPS- and IFN-γ-activated human microglial cells were exposed to lansoprazole or omeprazole, they displayed less toxicity towards neuroblastoma cells in culture [107]. Microglia may also perform protective functions, such as secretion of neurotrophic factors and the protective cytokines TGF-β and IL-10 [103], thus the role of PPIs on these functions deserves further study.

### Increased gastric pH

The basic function of all the compounds under discussion is to raise the gastric pH either directly or indirectly. Antacids act directly by neutralizing gastric acid while H2R antagonists and PPIs act by lessening acid production. Common antacids include calcium carbonate, magnesium carbonate, sodium bicarbonate or aluminum hydroxide, and like H2R antagonists and PPIs they are available over-the-counter in a number of preparations. Regardless of the mechanism by which the pH is increased, a lower level of stomach acid may have negative consequences for patients with MS. For example, there is a greater survival of bacteria in the stomach and small intestine following prolonged treatment with an acid suppressing agent [108-110]. Interestingly, a lower bacterial flora in the gastrointestinal tract was found to lessen the severity of EAE development [111]. The gut microflora has been shown to affect the innate immune response [112] and patients with MS have overactive neutrophils [97]. Increased neutrophil activity has been proposed to amplify and lengthen inflammation during an infection in patients with relapsing-remitting MS and may promote tissue injury and inflammation during MS [97]. Thus, a greater level of bacteria in the intestinal tract following a rise in gastric pH could, in theory, worsen the neutrophil response in MS. On the other hand, a rise in gastric pH has been associated with an increased risk of developing food allergies through promotion of Th2 responses [113-116] and the aluminum-based antacid sucralfate may enhance the Th2 effect [117,118]. In this example, a greater Th2 response due to a greater gastric pH would be predicted to lessen disease activity in MS.

When administered over long periods of time, agents that increase the gastric pH may lead to a deficiency of vitamin B12, particularly in older individuals [119,120]. Patients with MS can have low levels of vitamin B12 [121,122], suggesting that medications that block gastric acid production could be contributing to this deficient status.

## Conclusions

A large number of factors modulate the immune response during different phases of MS; treatment for dyspeptic pain is one factor that has the potential to affect the immune response. Managing gastric acid can be a recurrent issue faced over the lifetime of patients with MS. Although not proven, some interventions have the potential for disease aggravation while others would favor disease suppression or could be relatively neutral (Table 3). Although histamine release can result in inflammation, activation of the H2R is associated with immune suppression; administration of an H2R antagonist during a preexisting proinflammatory condition, such as occurs in MS, may lead to further immune stimulation. Thus, it is theoretically possible that H2R antagonists aggravate pathogenesis or lessen the effects of immunosuppressive drugs. An absence of overt changes in clinical signs following administration of H2R antagonists may not be sufficient to dismiss the potential negative effects of these drugs because much of the ongoing pathology can remain clinically silent. Besides H2R antagonists, PPIs and antacids can be administered for dyspeptic pain. In contrast to H2R antagonists, PPIs may have immunosuppressive properties, although they also can have unwanted side effects, for example, increased risk of gastric infection. Antacids as well as H2R antagonists and PPIs could also indirectly affect the immune system by enabling enhanced bacterial growth in the stomach and small intestine. Furthermore, prolonged use of inhibitors of gastric acid production might promote vitamin B12 deficiency, of which patients with MS appear to be at risk. We suggest that further investigations are warranted regarding the potential consequences of different approaches to the management of gastric acid in MS, especially over long periods of time with MS being a chronic condition.

**Table 3:** Summary of key effects of acid suppressing agents in relation to multiple sclerosis

Agent	Response
H2R antagonists	No reports of acute worsening of MS disease status Increases EAE incidence Promotes Th1 and Th17 responses Promotes production of proinflammatory cytokines Promotes T cell response, for example, proliferation Promotes suppression of Th2 response Block suppression of dendritic cells

PPIs	No reports of acute worsening of MS disease status Not studied in EAE Suppresses reactive oxygen species Lessens expression of adhesion molecules Suppresses production of proinflammatory cytokines

Increased gastric pH (H2R antagonists, PPIs, or antacids)	No reports of acute worsening of MS disease status Possibly alters microflora in the stomach and small intestine Possibly alters neutrophil response Can lead to vitamin B12 deficiency

EAE: experimental autoimmune encephalomyelitis; H2R: histamine receptor-2; MS: multiple sclerosis; PPI: proton pump inhibitor; Th: T helper cell.

## Abbreviations

BBB: blood-brain barrier; CNS: central nervous system; CSF: cerebrospinal fluid; DC: dendritic cell; EAE: experimental autoimmune encephalomyelitis; GERD: gastroesophageal reflux disease; H1R to H4R: histamine receptor-1 to -4; IFN: interferon; IL: interleukin; LPS: lipopolysaccharide; MRI: magnetic resonance imaging; MS: multiple sclerosis; PPI: proton pump inhibitor; TGF-β: transforming growth factor beta; Th: T helper cell; TNF: tumor necrosis factor.

## Competing interests

The authors declare that they have no competing interests.

## Authors' contributions

All authors contributed to writing the paper. All authors have read and approved the final version of the manuscript.

## Pre-publication history

The pre-publication history for this paper can be accessed here:

http://www.biomedcentral.com/1741-7015/10/57/prepub
